# Aberrant localization of FOXJ1 correlates with the disease severity and comorbidities in patients with nasal polyps

**DOI:** 10.1186/s13223-018-0296-z

**Published:** 2018-11-14

**Authors:** Yang Peng, Wei-jie Guan, Kai Sen Tan, Zhenchao Zhu, Zhuo Chen, Haiyu Hong, Zhaoni Wang, Tengfei Tian, Xiaoxue Zi, Yew Kwang Ong, Mark Thong, Li Shi, Qintai Yang, Qianhui Qiu, De-Yun Wang

**Affiliations:** 1 Department of Otolaryngology, Zhujiang Hospital, Southern Medical University, Guangzhou, Guangdong China; 2 Department of Otolaryngology, National University of Singapore, National University Health System, Singapore, Singapore; 3 State Key Laboratory of Respiratory Disease, National Clinical Research Center of Respiratory Disease, Guangzhou Institute for Respiratory Health, The First Affiliated Hospital, Guangzhou Medical University, Guangzhou, China; 4 Department of Otolaryngology Head & Neck Surgery, Guangdong General Hospital, Guangdong Academy of Medical Sciences, Guangzhou, Guangdong China; 5 Department of Otolaryngology Head & Neck Surgery, First Affiliated Hospital, Zhengzhou University, Zhengzhou, Henan China; 6 Department of Otolaryngology-Head and Neck Surgery, The 5th Affiliated Hospital, Sun Yat-sen University, Zhuhai, Guangdong China; 7 Department of Pediatrics, The 3rd Affiliated Hospital, Sun Yat-sen University, Guangzhou, Guangdong China; 8 Department of Otolaryngology Head and Neck Surgery, First Affiliated Hospital, Nanchang University, Nanchang, Jiangxi China; 9 Department of Otolaryngology, The Second Hospital, Shandong University, Jinan, China; 10 Department of Otorhinolaryngology-Head and Neck Surgery, The Third Affiliated Hospital, Sun Yat-Sen University, Guangzhou, Guangdong China; 11 Department of Otolaryngology, Yong Loo Lin School of Medicine, National University of Singapore, 1E Kent Ridge Road, Singapore, 119228 Singapore; 12 Department of Otolaryngology Head and Neck Surgery, Zhujiang Hospital, Southern Medical University, Guangzhou, 510282 Guangdong China

**Keywords:** Nasal polyps, Cilia, Forkhead-box J1, Mislocalization, Disease severity

## Abstract

**Background:**

Upper airway inflammatory diseases are associated with abnormal expression of nasal epithelial forkhead-box J1 (FOXJ1) which regulates motile cilia formation. We sought to investigate whether aberrant FOXJ1 localizations correlate with the disease severity and the co-existence of allergic rhinitis (AR) or asthma in patients with nasal polyps (NPs).

**Methods:**

We elucidated localization patterns of FOXJ1 by performing immunofluorescence assays in nasal specimens and cytospin samples from controls and patients with NPs. We also assayed mRNA expression levels of *FOXJ1* by using quantitative real-time polymerase chain reaction. Four localization patterns [normal (N), intermediate (I), mislocalization (M), and absence (A)] were defined. A semi-quantitative scoring system was applied for demonstrating FOXJ1 localization in five areas per paraffin section, with individual sections being scored between 0 and 2.

**Results:**

FOXJ1 localization score was significantly higher in samples from NPs than in controls (*P *< 0.001). Elevated FOXJ1 localization scores and down-regulation of *FOXJ1* mRNA levels were observed in NPs with co-existing AR or asthma (all *P *< 0.05). Moreover, FOXJ1 localization scores positively correlated with Lund–Mackay score (*r *= 0.362, *P *= 0.007). Of primary cytospin samples, the mean percentage of patients with FOXJ1 localization patterns N, I, M and A was 15.0%, 3.3%, 53.3% and 28.3% in NPs, and 82.5%, 5.0%, 5.0% and 7.5% in controls, respectively (*P *< 0.001).

**Conclusions:**

Aberrant localization of FOXJ1 correlates with the severity and co-existence of AR or asthma in patients with NPs, and might be a novel target for assessment and intervention in NPs.

**Electronic supplementary material:**

The online version of this article (10.1186/s13223-018-0296-z) contains supplementary material, which is available to authorized users.

## Background

Chronic rhinosinusitis (CRS) is characterized by chronic inflammation of the sinonasal mucosa, and may substantially impair the quality of life of affected patients. Based on the presence or absence of nasal polyps (NPs), CRS can be classified into CRS with NP (CRSwNP) and without NP (CRSsNP). To date, although the etiology and pathogenesis of NPs remain unclear, impaired ciliary function has been regarded as one of the pathophysiological characteristics of NPs [1, 2]. However, the correlation between ciliary impairment and the severity of NPs remains unclear. Additionally, it is unknown whether common comorbidities of NPs such as allergic rhinitis (AR) and asthma, in which impaired ciliary function is implicated, would further aggravate the impairment of ciliary function in NPs [3–5]. Elucidation of the pathogenic factors underlying NP formation (i.e. impaired mucociliary clearance) may facilitate better clinical management via early diagnosis and intervention.

Recently, we have revealed that the aberrant localization of dynein axonemal heavy chain 5 (DNAH5), a ciliary ultrastructural marker primarily responsible for primary ciliary dyskinesia (PCD), correlates with the disease severity and eosinophilia in patients with NPs [6]. Additionally, ciliary dysfunction and ultrastructural abnormalities of the bronchial epithelium have been closely associated with the pathogenesis of severe asthma [4]. These findings confirmed that chronic airway inflammatory diseases such as NPs are associated with dysfunctional ciliated cells lining the airway epithelium. Furthermore, increased centrosomal protein 110 (*CP110*) expression in airway mucosa from patients with NPs might have contributed to the poor ciliation of nasal epithelia, indicating the roles of functional impairment in driving the formation of defective cilia [7]. Therefore, further investigation is required to determine the effect of chronic airway inflammation on the formation of cilia and the mechanisms leading to the impairment of ciliary function in patients with NPs.

Forkhead transcription factor 1 (FOXJ1), also termed forkhead homologue 4, is a critical regulator in the maintenance of airway epithelial cell differentiation through the regulation of ciliogenesis [8]. Deletion and mutation of *Foxj1* result in motile ciliary disorders (i.e., defective ciliary development or assembly, abnormal ciliary structure-related gene expression) in mice, xenopus, and zebrafish, suggesting that *Foxj1* is critical to motile cilia formation [9, 10]. We have recently documented the down-regulation and aberrant localization of FOXJ1 in the inferior turbinate in patients with AR, which may be a crucial characteristic of the allergic nasal mucosa [11]. From the clinical perspective, NP is a common comorbidity of AR. To this end, we hypothesized that aberrant localization of FOXJ1 may be more prominent in patients with NPs compared with control subjects and that the aberrant localization of FOXJ1 may have accounted for the impaired cilia formation and motility in NPs. Patients with FOXJ1 mislocalization might have greater severity of NPs and may more frequently develop co-existing AR or asthma. By using immunofluorescence (IF) staining, we sought to unravel the association between the aberrant FOXJ1 localization with the severity of NPs and the presence of comorbidities (AR and asthma).

## Methods

### Patient recruitment

Study protocol approval was obtained from the institutional review boards of Guangdong General Hospital and the Second Hospital of Shandong University in China, and the National Healthcare Group Domain Specific Review Board of Singapore. All subjects provided informed consent.

Control subjects and patients with NPs were recruited from individual sites. CRSwNP diagnoses were made according to the *European Position Paper on Rhinosinusitis and Nasal Polyps 2012* [3], and AR was diagnosed according to *Allergic Rhinitis and its Impact on Asthma* (ARIA) [12] based on the clinical symptoms combined with skin prick test (*Dermatophagoides farinae*, *Dermatophagoides pteronyssinus*, pollens of oak, ragweed, mugwort, *Humulus japonicus*, cat and dog dander allergens) (Allergopharma, Reinbek, Germany) or serum total/specific IgE detected by using AllergyScreen^®^ (Mediwiss Analytic GmbH, Moers, Germany). The grades (G) of allergen-specific IgE were defined as follows: G0 (< 0.35 IU/ml), G1 (0.35–0.70 IU/ml), G2 (0.71–3.5 IU/ml), G3 (5.6–17.5 IU/ml), G4 (17.6–50 IU/ml), G5 (51–100 IU/ml) and G6 (> 100 IU/ml). The diagnosis of asthma was based on the history inquiry and physician’s diagnosis. Co-existence with asthma was deemed a more severe form of NPs [13]. The sinus opacity was scored radiologically and the severity and the size of NPs were assessed with the Lund–Mackay (LM) score [14]. Patients with antrochoanal polyps, fungal sinusitis, or susceptible PCD (an autosomal recessive congenital disease in which the structure and function of cilia are affected) were excluded. Control subjects were recruited from patients undergoing septoplasty due to anatomic variations and those who did not have allergic or inflammatory airway diseases, and biopsies were taken from the inferior turbinated mucosa (n = 40) during septal plastic surgery. Of these 40 control subjects, 20 were recruited for immunologic study and 34 for gene expression study (including 14 subjects for both assays). NP biopsies (n = 111) were performed during functional endoscopic sinus surgery. Among them, 83 subjects were recruited for immunologic study and 77 for gene expression study (including 39 subjects for both assays). Primary single-cell suspensions (1–2 × 10^5^ cells) were dissociated from control subjects (n = 4) and NPs (n = 6) for cytospin preparations. Details are shown in Table 1.

**Table 1:** Summary characteristics of the study participants

	Controls	Patients with CRSwNP
Sample size [no.]	40	111
Age, years (mean ± SD)	33.3 ± 10.7	42.6 ± 16.6
Gender (M/F)	25/15	79/32
AR [no. (%)]^a^	0 (0)	40 (36.0)
Asthma [no. (%)]^a^	0 (0)	28 (25.2)
Lund–Mackay CT scores (mean ± SD)^b^	NA	15.3 ± 4.4
Detection methods		
Paraffin specimens [no.]^c^	20	83
AR [no. (%)]	0 (0)	31 (37.3)
Asthma [no. (%)]	0 (0)	18 (21.7)
qRT-PCR [no.]	34	77
AR [no. (%)]	0 (0)	25 (32.5)
Asthma [no. (%)]	0 (0)	20 (26.0)
Cytospin preparations [no.]	4	6

*AR* allergic rhinitis, *CRSwNP* chronic rhinosinusitis with nasal polyp, *CT* computed tomography, *F* female, *M* male, *SD* standard deviation, *NA* not applicable

^a^None of the patients had co-existing allergic rhinitis with asthma been included

^b^CT scores were recorded in 54 patients with CRSwNP

^c^Hematoxylin–eosin staining, immunohistochemistry staining and immunofluorescence staining were performed in the same paraffin specimens from 20 control subjects and 83 patients with CRSwNP

### Cytospin preparation

Primary single-cell suspensions were harvested from nasal brushing (HydraFlock Flocked Swab, Puritan, Guilford, ME) or fresh nasal biopsy specimens. The specimens were initially treated with 10 mg/ml of Dispase II (Sigma-Aldrich, St. Louis, MO) at 4 °C overnight, followed by incubation with 1× trypspin/ethylenediaminetetraacetic acid at 37 °C for 15 min to acquire dissolved cells. Brushed or dissolved cells were subsequently fixed with 4% formaldehyde at room temperature for 10 min, followed by washing with 1× Dulbecco’s Phosphate Buffered Saline, and centrifugation at 1200 rpm for 5 min. Cytospin (1–2 × 10^4^ cells/slide) preparations were made at 500 rpm for 5 min with cytospin techniques (Shandon Cytospin 3 Cytocentrifuge, Thermo Fisher Scientific; Thermo Fisher Scientific, Waltham, MA).

### Immunohistochemistry staining

We performed hematoxylin–eosin staining, immunohistochemistry staining and evaluation of the epithelial structure as described previously [15]. Eosinophils were enumerated with hematoxylin–eosin staining, whereas neutrophils were stained with murine anti-human neutrophil elastase monoclonal antibodies (clone NP57; Dako A/S, Glostrup, Denmark) for immunohistochemistry staining. Epithelium with more than four layers was defined as having epithelial hyperplasia. The presence of stratified squamous epithelium was defined as squamous metaplasia. We evaluated the inflammatory cell types based on the proportions of eosinophils and neutrophils at 400× magnifications (high-power field, HPF), as described previously [15]. Briefly, the proportion of each type of cells in the 3 HPFs with respect to the total number of inflammatory cells in each HPF was calculated using the following formula: The proportion of the type of cells = (n1 + n2 + n3)/(m1 + m2 + m3) × 100%, where n1, n2, and n3 denoted the numbers of the type of inflammatory cells investigated in 3 HPFs, and m1, m2, and m3 denoted the total number of inflammatory cells in the 3 identical HPFs. All samples were coded confidentially and evaluated by two independent examiners by following the same protocol. NP with the percentage of eosinophils or neutrophils exceeding 10% was categorized as being eosinophilic or neutrophilic, respectively.

### IF staining

Protein expression of FOXJ1 and acetylated alpha-tubulin was examined by using IF staining on paraffin sections and primary single-cell cytospin sections of nasal biopsies. The sections were incubated with primary polyclonal antibodies [rabbit anti-human FOXJ1 [HPA005714] (Sigma, Ronkonkoma, NY) and mouse anti-human acetylated alpha tubulin [clone ab24610] (Abcam, Cambridge, MA)] overnight at 4 °C, followed by incubation with Alexa Fluor 488- or Alexa Fluor 594-conjugated secondary antibodies (Life Technologies, Carlsbad, CA) at 37 °C for 1 h.

Cellular nuclei were visualized by staining with 4′ 6-diamidino-2-phenylindole (DAPI) (Life Technologies, Carlsbad, CA). For negative controls, primary antibodies were substituted with species- and subtype-matched antibodies of the same concentration. Images were acquired with fluorescence microscopy (Olympus IX51, Tokyo, Japan).

### Evaluation of FOXJ1 localization

To elucidate the different localization patterns, FOXJ1 (red) and acetylated alpha tubulin (green) were evaluated according to a semi-quantitative scoring system. The nuclei were stained with DAPI (blue). By following our recently published study [11], the localization of FOXJ1 expression was defined as follows: Presence of FOXJ1 in the nuclei only (Normal; N); Presence of FOXJ1 in both the nuclei and axoneme (Intermediate; I); Presence of FOXJ1 within the axoneme only (Mislocalization; M); Absence of FOXJ1 in the nuclei and axoneme (Absence; A).

We randomly selected five areas of cilia staining from each paraffin sample in a blinded manner to elucidate the localization of FOXJ1 at 400× magnification. Based on the criterion proposed by Shoemark et al. [16] and our recently published study [11], each area was assigned a score between 0 and 2, with 0 corresponding to the field containing > 70% N-localization; 1 for > 70% I + N-localization; and 2 for ≥ 30% with M- or A-localization. Each field was scored by two reviewers blinded to the grouping. We then averaged the score of five areas for subsequent reporting. We randomly selected single ciliated cells from primary specimens for cytospin preparations, and the localization pattern of individual cell was evaluated at 1000× magnification under oil immersion lens.

### Quantitative real-time polymerase chain reaction

The mRNA expression levels of *FOXJ1* were validated with quantitative real-time polymerase chain reaction (PCR) (SYBR Green, Promega, Madison, WI, USA) in accordance with the “The Minimum Information for Publication of Quantitative Real-Time PCR Experiments (MIQE) guidelines” (Additional file 1: Table S2) [17]. The relative gene expression was calculated using the 2^−ΔΔCt^ method normalized against the housekeeping gene, glyceraldehyde 3-phosphate dehydrogenase (GAPDH). Selection of the appropriate reference gene depends on the individual experimental setting. Despite the use of a single reference gene, we have confirmed the suitability of *GAPDH* as the reference gene in our previous studies [18, 19]. Amplification of *FOXJ1* and *GAPDH* was performed with the following primers: *FOXJ1* forward (5′-GTGAAGCCTCCCTACTC-3′), *FOXJ1* reverse (5′-AATTCTGCCAGGTGGG-3′) (PrimerPair ID: H_FOXJ1_1, Gene ID: 2302; Sigma-Aldrich, St. Louis, MO); *GAPDH* forward (5′-ACAGTTGCCATGTAGACC-3′), *GAPDH* reverse (5′-TTT TTGGTTGAGCACAGG-3′) (PrimerPair ID: H_GAPDH_1, Gene ID: 2597; Sigma-Aldrich, St. Louis, MO).

### Statistical analysis

Statistical analyses were conducted with SPSS software (version 18.0, SPSS, Chicago, IL, USA). Mann–Whitney two-sided non-parametric test was applied to compare FOXJ1 localization score and *FOXJ1* mRNA expression levels in paraffin-embedded specimens. The Fisher’s exact test was employed to assess for the differences in FOXJ1 localization patterns between control subjects and patients with NPs for cytospin specimens. *P *< 0.05 was deemed statistically significant for all analyses.

## Results

### Subject characteristics

The clinical characteristics of control subjects and patients with NPs are summarized Table 1. For paraffin specimens, all CRSwNP patients with AR (37.3%, 31/83) were diagnosed with AllergyScreen^®^, and were graded as having G1 (n = 3), G2 (n = 11), G3 (n = 4) and G4 (n = 13) AR (Additional file 1: Table S1). The hematoxylin–eosin staining showed that 90.4% (75/83) of specimens from NPs displayed varying degrees of epithelial remodeling.

### Increased aberrant localization of FOXJ1 in NPs

**Fig. 1:**
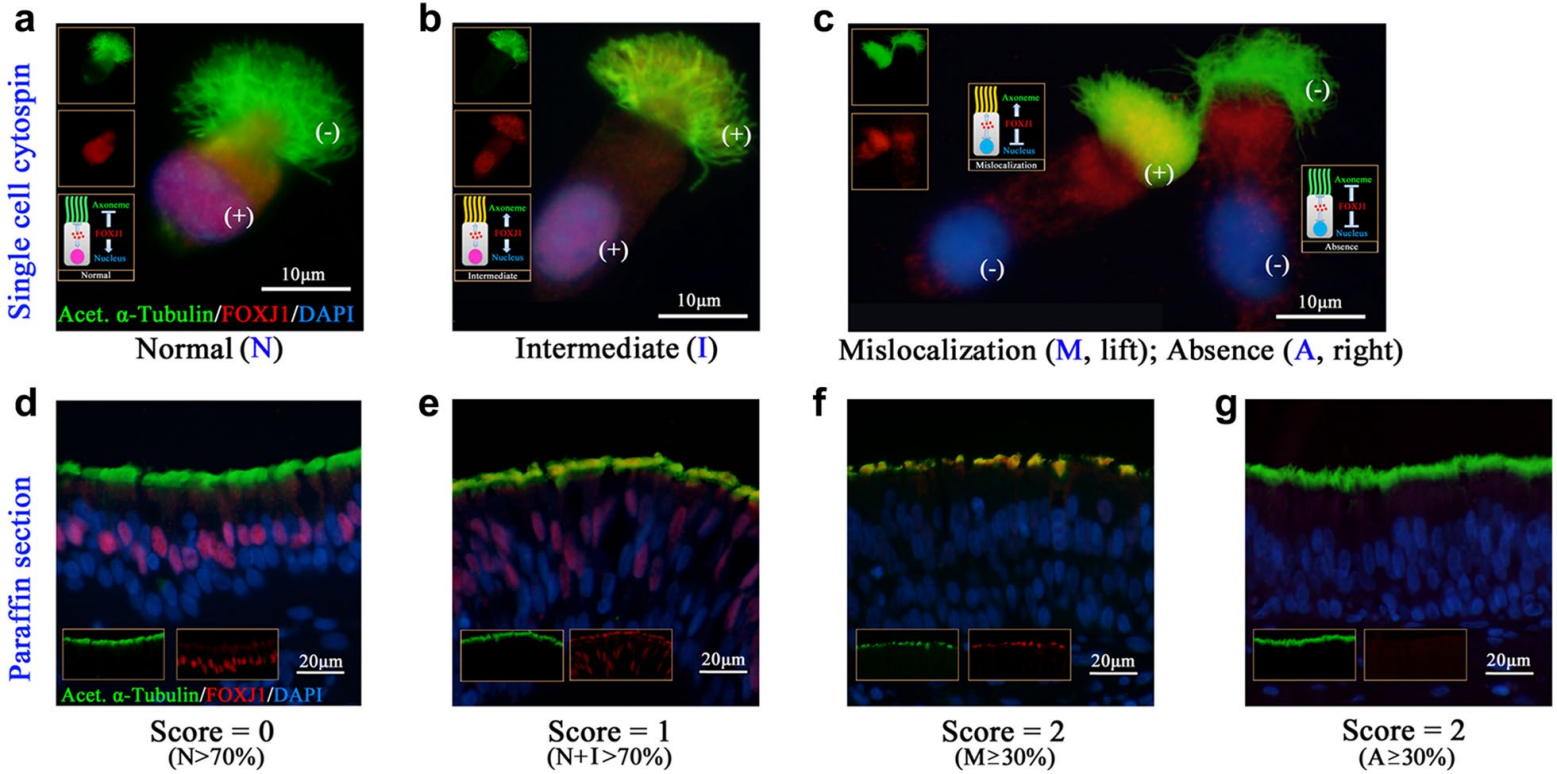
Four distinct localization patterns of FOXJ1 in single cells and paraffin specimens. Four localization patterns are observed in single cells (**a**–**c**) and paraffin specimens (**d**–**g**). FOXJ1 grading system: Score 0 = (N > 70%) (**d**); Score 1 = (N + I > 70%) (**e**); Score 2 = (M or A ≥ 30%) (**f**, **g**). (+): positive staining of FOXJ1; (−): negative staining of FOXJ1. Acet. α-Tubulin: Acetylated α-Tubulin; DAPI: 4′,6-diamidino-2-phenylindole; FOXJ1: forkhead-box J1

**Fig. 2:**
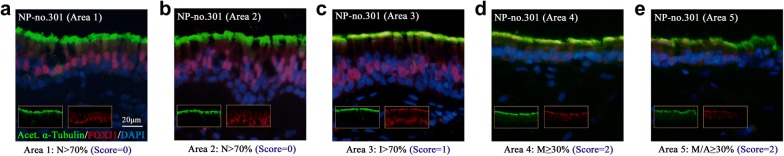
A semi-quantitative scoring system for elucidating FOXJ1 localization under fluorescence microscopy. We elucidated the FOXJ1 localization score of nasal polyps from a patient (ID: NP-no. 301) by using our scoring system based on five areas under microscopy. The individual score of the 5 areas was 0 (**a**), 0 (**b**), 1 (**c**), 2 (**d**), 2 (**e**); and the average score was (0 + 0+1 + 2+2)/5 = 1. FOXJ1: forkhead-box J1; NP: nasal polyp

We initially examined the localization patterns of FOXJ1 in nasal epithelial cilia by performing IF staining of samples from control subjects and patients with NPs. As shown in Fig. 1, four distinct localization patterns (N, I, M and A, Fig. 1a–c) were observed in isolated primary single cells. Based on our semi-quantitative FOXJ1 scoring system (Fig. 1d–g), we scored five areas and obtained the average score of each paraffin sections (Fig. 2a–e).

**Fig. 3:**
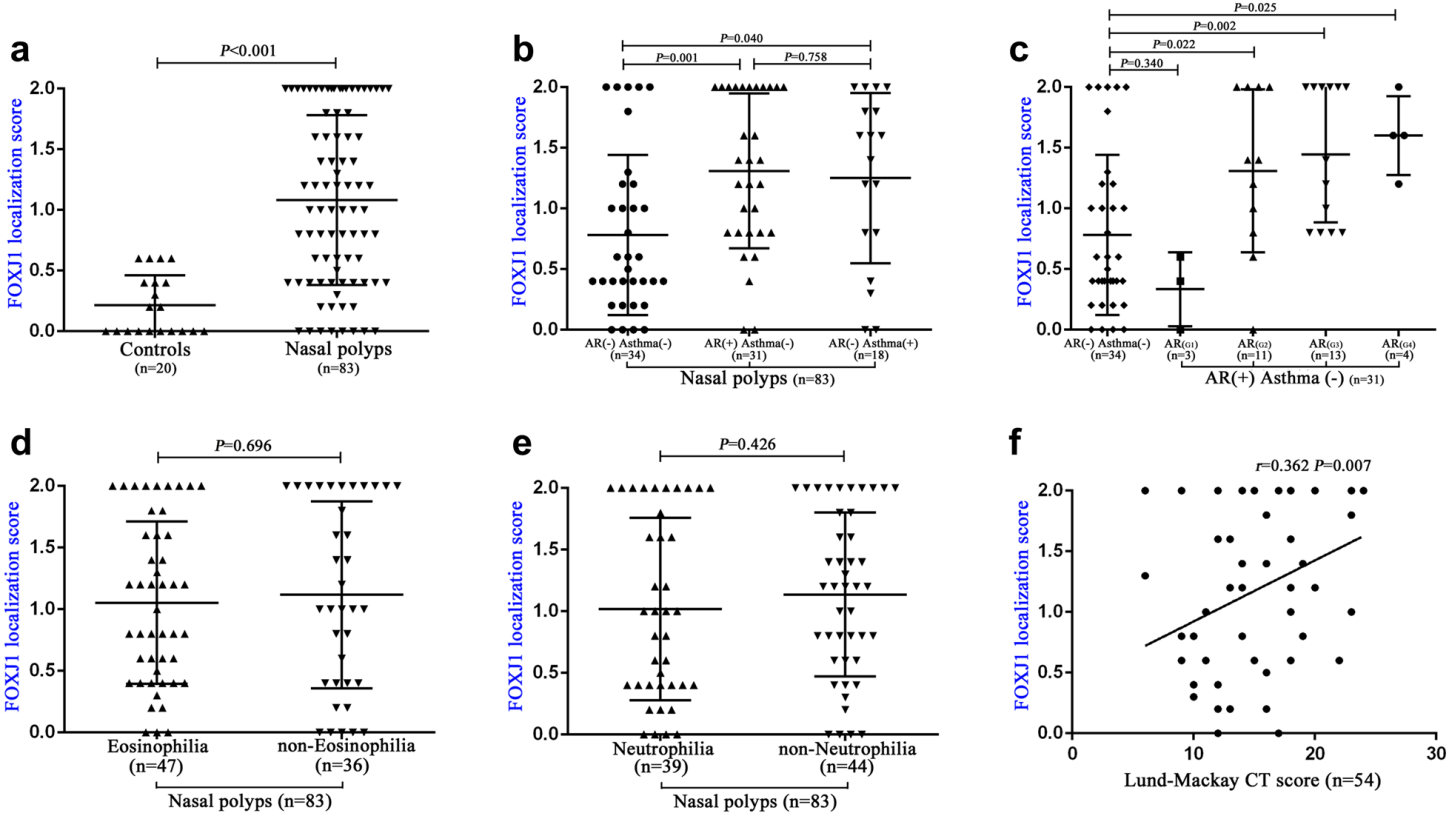
FOXJ1 localization score correlates with disease severity and co-existence of allergic rhinitis or asthma in patients with nasal polyps. FOXJ1 localization score between controls and patients with nasal polyps (**a**), and subgroup analysis (**b**, **c**). Correlation between FOXJ1 localization score and eosinophilia (**d**), neutrophilia (**e**), and Lund–Mackay scores (**f**, fifty-four CT scores in patients with nasal polyps were recorded.). FOXJ1: forkhead-box J1

The median (the 1st and 3rd quartile) score was 1.00 (0.40, 1.80) for samples from NPs (n = 83), and 0.10 (0, 0.40) for samples from control subjects (n = 20) (*P *< 0.001, Fig. 3a). We also evaluated 10 single cilia cells from each primary samples from cytospin preparations. The N, I, M and A pattern was observed in 15.0% (9/60), 3.3% (2/60), 53.3% (32/60) and 28.3% (17/60) of ciliated cells in patients with NPs (n = 6), respectively. The corresponding percentage was 82.5% (33/40), 5.0% (2/40), 5.0% (2/40), and 7.5% (3/40) in control subjects (n = 4), respectively (*P *< 0.001, Table 2).

**Table 2:** Summary of FOXJ1 localization in primary cells cytospin

	Controls	CRSwNP patients	*P* value
Primary cells specimens [no.]	4	6	NA
Single ciliated cells evaluated [no.]^a^	40	60	NA
FOXJ1 localization [no. (%)]	–	–	< 0.001
Normal	33 (82.5)	9 (15.0)	–
Intermediate	2 (5.0)	2 (3.3)	–
Mislocalization	2 (5.0)	32 (53.3)	–
Absence	3 (7.5)	17 (28.3)	–

*CRSwNP* chronic rhinosinusitis with nasal polyps, *FOXJ1* forkhead-box J1, *NA* not applicable

^a^Ten single cilia cells were selected randomly from each primary cell specimen

### FOXJ1 localization score correlates with the presence of AR and asthma in patients with NPs

We interrogated if there were major differences between FOXJ1 localization score and the co-existence of AR or asthma in patients with NPs. Note that patients with co-existing AR and asthma have been excluded from our study. The median (1st, 3rd quartile) localization score was 0.55 (0.40, 1.15) for NPs with neither AR nor asthma (n = 34), 1.40 (0.80, 2.00) for NPs with isolated AR (n = 31), and 1.50 (0.80, 1.80) for NPs with isolated asthma (n = 18). The FOXJ1 localization score was significantly increased in both NPs with isolated AR, and NPs with isolated asthma (both *P *< 0.05, Fig. 3b).

Next, we examined the difference and association of the FOXJ1 localization score and the grading (G) of allergen-specific IgE in patients with NPs and AR. Compared with NPs without AR or asthma, the FOXJ1 localization score was significantly increased in patients with NPs who had more greater degree of allergy (G2-G4) (*P *< 0.05, Fig. 3c). However, the FOXJ1 localization score did not differ significantly between NPs and those with minor allergy (G1) (*P *> 0.05, Fig. 3c).

### FOXJ1 localization score correlates with the severity, but not the inflammatory phenotypes, of NPs

In our study, the percentage of eosinophils or neutrophils exceeding 10% was categorized as eosinophilia or neutrophilia [15, 20]. Of these, 56.6% (47/83) of NPs were eosinophilic and 47.0% (39/83) were neutrophilic, whereas 14 specimens have mixed eosinophilic and neutrophilic inflammatory phenotypes. We sought to determine if there were underlying associations between FOXJ1 localization score and the presence of eosinophilic or neutrophilic airway inflammation. The median (1st, 3rd quartile) FOXJ1 localization score was 1.00 (0.45, 1.60) for eosinophilic NPs (n = 47) and 1.00 (0.40, 2.00) for non-eosinophilic NPs (n = 36) (*P *> 0.05, Fig. 3d). When stratified according to the presence of neutrophilia only, we also noted no marked difference in the median (1st and 3rd quartile) FOXJ1 localization score between neutrophilic (n = 39) and non-neutrophilic NPs (n = 44) [1.00 (0.40, 1.90) vs. 1.20 (0.60, 1.80), *P *> 0.05, Fig. 3e).

The Lund–Mackay scores were available in 54 patients with NPs. The FOXJ1 localization score positively correlated with the Lund–Mackay CT score (*r *= 0.362, *P *= 0.007, Fig. 3f). The finding confirms that FOXJ1 localization score is useful for predicting NP severity and presence of comorbidities, but does not distinguish between the inflammatory phenotypes.

### Altered FOXJ1 mRNA expression in NPs and its comorbidities

**Fig. 4:**
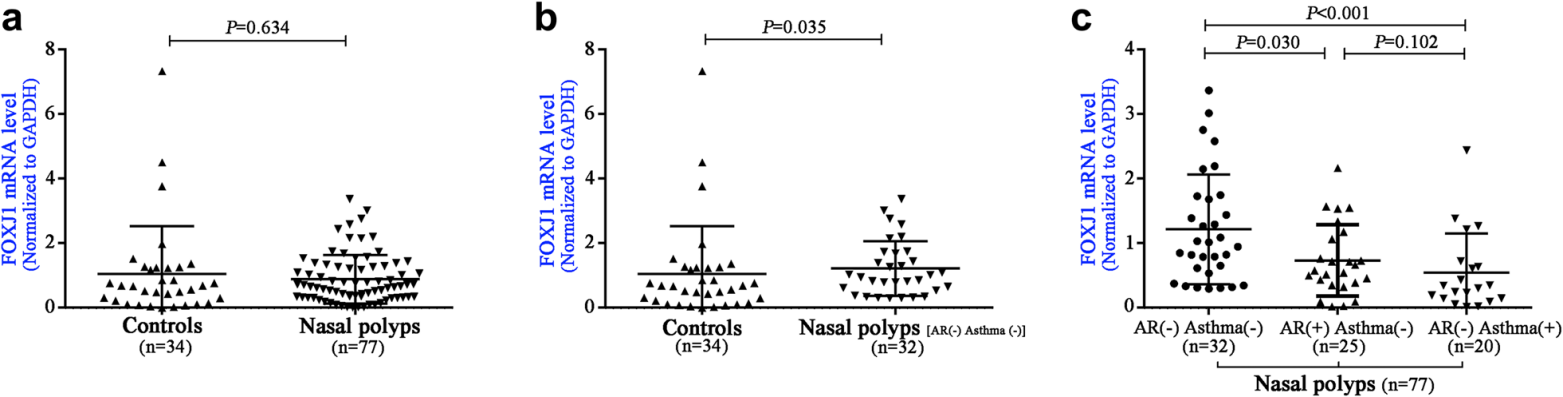
The mRNA expression levels of *FOXJ1* between controls and patients with nasal polyps. No significant difference of FOXJ1 mRNA expression levels was found between controls and patients with NPs (n = 77) (**a**). The FOXJ1 mRNA expression levels were found to be higher in NPs without AR or asthma (n = 32) than control subjects (**b**). The FOXJ1 mRNA expression levels were significantly downregulation in both NPs with isolated AR (n = 25), and NPs with isolated asthma (n = 20) (**c**). AR: allergic rhinitis; FOXJ1: forkhead-box J1; NPs: nasal polyps

We compared *FOXJ1* gene expression with quantitative real-time PCR from controls (n = 34) and patients with NPs (n = 77). We initially evaluated the *FOXJ1* mRNA expression levels in patients with NPs (n = 77) compared with controls. However, *FOXJ1* mRNA expression levels did not differ statistically (*P *= 0.634, Fig. 4a). However, the *FOXJ1* mRNA expression levels were markedly higher in NPs without AR or asthma (n = 32) than in control subjects (*P *= 0.035, Fig. 4b). Furthermore, the *FOXJ1* mRNA expression levels were significantly down-regulated in NPs with isolated AR (n = 25), and NPs with isolated asthma (n = 20) (both *P *< 0.05, Fig. 4c).

## Discussion

The upper airways play an important role in removing airborne pathogens and allergens via effective mucociliary clearance, which is essential to maintaining proper nasal epithelial defense. Although most studies have focused on congenital (or genetic) ciliary dysfunction in PCD, acquired ciliary dysfunction also contributes substantially to the pathogenesis of chronic airway inflammatory diseases such as asthma, AR and CRS, possibly due to the negative impacts of chronic inflammation on mucociliary clearance [4, 21, 22]. As a master ciliogenesis transcription factor, *FOXJ1* is critical to the regulation of ciliary differentiation and mucociliary clearance, and is essential for the assembly of motile cilia in vertebrates through the regulation of genes that are specific to motile cilia or necessary for basal body apical transport [10, 23]. Notably, our study has demonstrated markedly aberrant FOXJ1 localization and the down-regulation of *FOXJ1* mRNA expression levels in patients with NPs who have co-existing AR or asthma, which strongly suggested that aberrant FOXJ1 localization might have contributed considerably to the greater disease severity in patients with NPs. Nonetheless, the method for detecting *FOXJ1* gene expression could be improved because the MIQE guideline has endorsed the use of more than one reference gene [17].

Our previous study has demonstrated that *FOXJ1* mRNA expression levels were markedly higher in surgically resected tissues from patients with NPs than those from controls [19]. Moreover, we have recently reported that the increased aberrant localization of FOXJ1 protein and down-regulation of *FOXJ1* mRNA expression in allergic nasal mucosa [11]. These two findings are similar to the principal findings of the present study (Fig. 4b, c). In addition, the pattern of FOXJ1 localization partially resembled our previous findings. That is, more aberrant localization of DNAH5 correlated with the Lund–Mackay score in patients with NPs [6]. Intriguingly, FOXJ1 localization score did not correlate with eosinophilic inflammation in NPs, which contrasted with the positive correlation between DNAH5 localization score and eosinophilic airway inflammation [6]. It remains elusive whether eosinophilic and neutrophilic inflammation confers equivalent impacts on the mislocalization or absence of FOXJ1 in nasal epithelial ciliated cells in patients with NPs. Therefore, the cause of aberrant localization of FOXJ1 and DNAH5 we observed in NPs needs to be further determined.

Our study has important clinical implications. Our findings suggested that impaired mucociliary clearance as a consequence of the aberrant expression of ciliogenesis markers might represent a common pathway linking to the pathogenesis of upper airway diseases such as AR, asthma and NPs. FOXJ1 localization might be an important metric which should be incorporated in future clinical assessment of mucociliary clearance and prediction of concomitant diseases complicating the management of NPs. Moreover, the aberrant localization of FOXJ1 might represent a novel therapeutic target because the commercially available drugs such as Gelomyrtol Forte™ are capable of normalizing FOXJ1 expression in vitro [24]. Therefore, surgery alone for NPs with co-existing severe AR and asthma may not be sufficient to completely reverse the course of disease in which markedly impaired mucociliary function is implicated. Future management of NPs might benefit from the incorporation of mucociliary function and more cutting-edge approaches (i.e. inhaled viral vectors and gene editing with CRSPIR–Cas9) for rectifying ciliopathy in chronic airway diseases that fail to respond to conventional anti-inflammatory or antibiotic therapy.

A major caveat was that we cannot address whether the aberrant localization of FOXJ1 may be directly responsible for the aberrant localization of ciliary ultrastructural markers associated with the outer/inner dynein arms and radial spokes that can also regulate ciliary beating. The causality of aberrant *FOXJ1* expression and the severity of NPs as well as the co-existence of AR and asthma cannot be disentangled with the present study design. The association between FOXJ1 mislocalization and the function of respiratory cilia cannot be addressed. We did not perform scanning or transmission electron microscopy to confirm the ultrastructural defects nor did we directly observe the whole course of ciliogenesis in NPs. In addition, we did not examine the correlation between the aberrant localization of FOXJ1 and mucociliary function (e.g. nasal nitric oxide levels, saccharine test). Finally, although sinus CT or the assessment of comorbidity are clinically valuable approaches for NP evaluation, these tests failed to evaluate nasal epithelium from a pathologic perspective and are thus not specific to ciliary assessment. Nonetheless we believe that our findings will inspire ongoing studies to delineate the association between impaired mucociliary clearance and NPs as well as the exploration of targeted therapies.

In summary, the aberrant localization of FOXJ1 correlates with the disease severity and the co-existence of AR and asthma in patients with NPs, which may provide novel candidate targets for more accurate clinical assessment and future treatment guidelines for NPs management.

## Additional file

**Additional file 1: Table S1.** Summary of characteristics from CRSwNP patients co-existence with AR elucidated by using IF staining. **Table S2.** dMIQE checklist for demonstrating the methods to perform quantitative polymerase chain reaction.

## Abbreviations

AR: allergic rhinitis
ARIA: Allergic Rhinitis and its Impact on Asthma
CRS: chronic rhinosinusitis
CT: computed tomography
DAPI: 4′ 6-diamidino-2-phenylindole
DNAH5: dynein axonemal heavy chain 5
FOXJ1: forkhead-box J1
IF: immunofluorescence
NP: nasal polyp
MIQE: The Minimum Information for Publication of Quantitative Real-Time PCR Experiments
PCD: primary ciliary dyskinesia
PCR: polymerase chain reaction
