# Supplementary material for: Aberrant localization of FOXJ1 correlates with the disease severity and comorbidities in patients with nasal polyps
Source: Allergy Asthma Clin Immunol. 2018 Nov 14;14:71. doi: 10.1186/s13223-018-0296-z (PMC6234688; doi:10.1186/s13223-018-0296-z)
Supplement: Supplementary file 1 — Additional file 1: Table S1. Summary of characteristics from CRSwNP patients co-existence with AR elucidated by using IF staining. Table S2. dMIQE checklist for demonstrating the methods to perform quantitative polymerase chain reaction. [file 13223_2018_296_MOESM1_ESM.docx]

**Additional file**

**TABLE S1. Summary characteristics of CRSwNP patients with co-existign allergic rhinitis whose samples were subject to immunofluorescence staining ***

| **No.** | **Age, yr** | **Gender** | **Specific-IgE, IU/ml (Allergen to†)** | **Grade of atopy** | **Total IgE, IU/ml** |
| --- | --- | --- | --- | --- | --- |
| 1 | 67 | F | 3.8 (Tree mixture) | 3 | 99.6 |
| 2 | 73 | F | 29 (*D. pteronyssinus*) | 4 | 84.3 |
| 3 | 24 | F | 3.6 (*D. pteronyssinus*) | 3 | 8356 |
| 4 | 9 | M | 0.6 *(D. pteronyssinus*) | 1 | 313.8 |
| 5 | 59 | M | 1.2 (Pigweed) | 2 | 1443 |
| 6 | 15 | M | 17.2 (Pigweed) +12.9 (Weed mixture) | 3 | 349 |
| 7 | 52 | M | 23.4 (Tree mixture) | 4 | 15.3 |
| 8 | 28 | M | 7.1 (*D. pteronyssinus*) | 3 | 3278 |
| 9 | 69 | M | 1.2 (Mould fungi mixture) | 2 | 66.7 |
| 10 | 20 | M | 2.9 (Mould fungi mixture) | 2 | 35.9 |
| 11 | 41 | M | 3 (*D. pteronyssinus*) | 2 | 65540 |
| 12 | 54 | M | 2.9 (Mould fungi mixture) | 2 | 117.5 |
| 13 | 15 | F | 0.5 (Mould fungi mixture) | 1 | 18.6 |
| 14 | 42 | M | 0.6 (*D. pteronyssinus*) | 1 | 796.3 |
| 15 | 70 | M | 11.8 (Mould fungi mixture) + 2.9 (*D. pteronyssinus*) | 3 | 5742 |
| 16 | 24 | M | 8.2 (*D. pteronyssinus*) | 3 | 217 |
| 17 | 33 | M | 1.6 (*D. pteronyssinus*) + 0.5 (Tree mixture) | 2 | 334.3 |
| 18 | 18 | M | 3.4 (*D. pteronyssinus*) | 2 | 105.8 |
| 19 | 30 | M | 1.7 (*D. pteronyssinus*) | 2 | 65540 |
| 20 | 61 | F | 1.6 (*D. pteronyssinus*) | 2 | 249.2 |
| 21 | 30 | M | 4 (*D. pteronyssinus*) | 3 | 185.7 |
| 22 | 62 | F | 4.5 (Mould fungi mixture) | 3 | 11540 |
| 23 | 37 | F | 5.4 (*D. pteronyssinus*) + 5.4 (Mould fungi mixture) | 3 | 459.7 |
| 24 | 38 | M | 4.4 (*D. pteronyssinus*) | 3 | 12.5 |
| 25 | 58 | M | 5.2 (*D. pteronyssinus*) | 3 | 285.2 |
| 26 | 26 | M | 6.9 (*D. pteronyssinus*) | 3 | 157.7 |
| 27 | 35 | M | 41.4 (*D. pteronyssinus*) | 4 | 1310 |
| 28 | 45 | M | 3.5 *(D. pteronyssinus*) | 2 | 726.2 |
| 29 | 22 | M | 27.4 (Tree mixture) | 4 | 121.7 |
| 30 | 34 | M | 5.7 (Tree mixture) | 3 | 168 |
| 31 | 30 | M | 1.1 (*D. pteronyssinus*) | 2 | 473.2 |

* AR diagnosed was on the basis of a thorough history, physical examination, and serum specific IgE by using AllergyScreen^®^ test (n=31).

† The AllergyScreen^®^ test include the following aeroallergens: D. pteronyssinus, pigweed, mould fungi mixture, tree mixture, house dust, weed mixture, dog epithelium and dander, cat epithelium and dander, cockroach, mulberry.

AR: allergic rhinitis; CRSwNP: chronic rhinosinusitis with nasal polyp; D. pteronyssinus: Dermatophagoides pteronyssinus; F: female; IF: Immunofluorescence; M: male

**Table S2. dMIQE checklist for demonstrating the methods to perform quantitative polymerase chain reaction.**

| **ITEM TO CHECK** | **IMPORTANCE** | **DESCRIPTION / WHERE?** |
| --- | --- | --- |
| **EXPERIMENTAL DESIGN** |  |  |
| Definition of experimental and control groups | E | See **Materials and Methods** section |
| Number within each group | E | See **Materials and Methods** section |
| Assay carried out by core lab or investigator's lab? | D | Investigator’s lab |
| Acknowledgement of authors' contributions | D | See **Author’s contribution** section |
| **SAMPLE** |  |  |
| Description | E | RNA extracted from inferior turbinate (controls) or nasal polyps (patients) |
| Volume/mass of sample processed | D | 10-50 mg |
| Microdissection or macrodissection | E | Macrodissection |
| Processing procedure | E | The tissues were collected and immediately incubated with RNAlater (Sigma-Aldrich Inc.) within 1 hour after surgical resection from the patients with NPs. We incubated the samples at 2-8℃ for overnight, and transferred to the -80℃ freezers for storage. |
| If frozen - how and how quickly? | E | Not applicable. |
| If fixed - with what, how quickly? | E | The tissues were collected and immediately incubated with RNAlater (Sigma-Aldrich Inc.) within 1 hour after surgical resection from the patients with NPs. |
| Sample storage conditions and duration (especially for FFPE samples) | E | After fixation with RNAlater, samples were incubated at 2-8℃ for overnight, and transferred to the -80℃ freezers for storage. |
| **NUCLEIC ACID EXTRACTION** |  |  |
| Procedure and/or instrumentation | E | Total RNA was extracted from frozen nasal epithelial tissues in RNAlater using the mirVana miRNA Isolation Kit (Life Technologies). |
| Name of kit and details of any modifications | E | MirVana miRNA Isolation Kit (Life Technologies) and protocol supplied by manufacturer. |
| Source of additional reagents used | D | Not applicable. |
| Details of DNase or RNAse treatment | E | No treatment. |
| Contamination assessment (DNA or RNA) | E | Spectrophotometric analysis with measurements at 320 nm, 280 nm and 260 nm. |
| Nucleic acid quantification | E | Assessment based on the ratio of 260 nm/280 nm |
| Instrument and method | E | Nanodrop©2000 (Thermo Scientific) |
| Purity (A260/A280) | D | > 1.9 and <2.1 |
| Yield | D | Variable, depending on the day of the developmental stage we investigated. |
| RNA integrity method/instrument | E | Not applicable. |
| RIN/RQI or Cq of 3' and 5' transcripts | E | Not applicable. |
| Electrophoresis traces | D | Not applicable. |
| Inhibition testing (Cq dilutions, spike or other) | E | Not performed. |
| **REVERSE TRANSCRIPTION** |  |  |
| Complete reaction conditions | E | 1 μg of total RNA was reverse transcribed into cDNA using Maxima Reverse Transcriptase Kit (Thermo Fisher Scientific) in 20 μl reaction volumes according to manufacturer’s protocol. |
| Amount of RNA and reaction volume | E | 1 μg of total RNA per 20 μl reaction volume for each reverse transcription reaction |
| Priming oligonucleotide (if using GSP) and concentration | E | Reaction Mix (4 μl Reaction Mix per 20 μl reaction volume) from Maxima Reverse Transcriptase Kit (Thermo Fisher Scientific) contains the remaining reaction components: reaction buffer, dNTPs, oligo (dT) 18 and random hexamer primers. |
| Reverse transcriptase and concentration | E | Maxima Enzyme Mix (2 μl Maxima Enzyme Mix per 20 μl reaction volume) from Maxima Reverse Transcriptase Kit (Thermo Fisher Scientific). |
| Temperature and time | E | Incubation for 30 min at 25°C followed by 15 min at 50°C, and termination of the reaction by heating at 85°C for 5 min. |
| Manufacturer of reagents and catalogue numbers | D | Maxima Reverse Transcriptase Kit (Thermo Fisher Scientific, #K1642) |
| Cqs with and without RT | D | Not applicable. |
| Storage conditions of cDNA | D | At -20°C in sterile test tubes |
| **qPCR TARGET INFORMATION** |  |  |
| If multiplex, efficiency and LOD of each assay. | E | Not multiplex. |
| Sequence accession number | E | See **Materials and Methods** section. |
| Location of amplicon | D | - |
| Amplicon length | E | 90bp (GAPDH), 144bp (FOXJ1) |
| *In silico* specificity screen (BLAST, etc) | E | This has been applied: http://www.ncbi.nlm.nih.gov/tools/primer-blast/index.cgi?LINK_LOC=BlastHome as target organism |
| Pseudogenes, retropseudogenes or other homologs? | D | - |
| Sequence alignment | D | - |
| Secondary structure analysis of amplicon | D | - |
| Location of each primer by exon or intron (if applicable) | E | Both the primers of *GAPDH* and *FOXJ1* were based on the single exon |
| What splice variants are targeted? | E | Not applicable. |
| **qPCR OLIGONUCLEOTIDES** |  |  |
| Primer sequences | E | See **Materials and Methods** section. |
| RTPrimerDB Identification Number | D | - |
| Probe sequences | D | - |
| Location and identity of any modifications | E | No modifications. |
| Manufacturer of oligonucleotides | D | - |
| Purification method | D | - |
| **qPCR PROTOCOL** |  |  |
| Complete reaction conditions | E | 95 °C for 10 min as an initial step, followed by 40 cycles of 95 °C for 15 s and 60 °C for 60 s, and termination with the melting-curve analysis at 95°C for 15s, 60°C for 60s and 95°C for 15 s to discriminate the main reaction products from other nonspecific ones or primer-dimers. |
| Reaction volume and amount of cDNA/DNA | E | 20 ng of cDNA in 20 μl reaction volume |
| Primer, (probe), Mg++ and dNTP concentrations | E | Manufacturers’ proprietary [GoTaq® qPCR Systems (Promega)]. |
| Polymerase identity and concentration | E | Manufacturers’ proprietary [GoTaq® qPCR Systems (Promega)]. |
| Buffer/kit identity and manufacturer | E | GoTaq® qPCR Systems (Promega) |
| Exact chemical constitution of the buffer | D | - |
| Additives (SYBR Green I, DMSO, etc.) | E | SYBR Green I (Promega) |
| Manufacturer of plates/tubes and catalog number | D | - |
| Complete thermocycling parameters | E | 95 °C for 10 min as an initial step followed by 40 cycles of 95 °C for 15 s and 60 °C for 60 s, and ending with melting-curve analysis at 95°C for 15s, 60°C for 60s and 95°C for 15 s. |
| Reaction setup (manual/robotic) | D | - |
| Manufacturer of qPCR instrument | E | Applied Biosystems ViiA™ 7 Real-Time PCR System |
| **qPCR VALIDATION** |  |  |
| Evidence of optimisation (from gradients) | D | - |
| Specificity (gel, sequence, melt, or digest) | E | Melting curves for each biological replicate. |
| For SYBR Green I, Cq of the NTC | E | > 40 or no amplification |
| Standard curves with slope and y-intercept | E | Not applicable [we performed the 2^(-ΔΔCt) method which is based on normalization with a single reference gene.]. |
| PCR efficiency calculated from slope | E | Not applicable. |
| Confidence interval for PCR efficiency or standard error | D | - |
| r2 of standard curve | E | Not applicable. |
| Linear dynamic range | E | Not applicable. |
| Cq variation at lower limit | E | Not applicable. |
| Confidence intervals throughout range | D | - |
| Evidence for limit of detection | E | Not applicable. |
| If multiplex, efficiency and LOD of each assay. | E | Not applicable. |
| **DATA ANALYSIS** |  |  |
| qPCR analysis program (source, version) | E | QuantStudio™ Real-Time PCR Software v1.1 (Life technologies). |
| Cq method determination | E | Manual, set for all assays at ΔRn=0.04. |
| Outlier identification and disposition | E | No outliers identified. |
| Results of NTCs | E | Negative. |
| Justification of number and choice of reference genes | E | We employed *GAPDH* as the reference gene for normalization. |
| Description of normalization method | E | We employed the 2^(-ΔΔCt) algorithm. |
| Number and concordance of biological replicates | D | - |
| Number and stage (RT or qPCR) of technical replicates | E | For most cases 3 biological replicates were used and in some cases 6 biological replicates were used. |
| Repeatability (intra-assay variation) | E | Depending on the transcript analyzed |
| Reproducibility (inter-assay variation, %CV) | D | - |
| Power analysis | D | - |
| Statistical methods for result significance | E | See **Materials and Methods** section |
| Software (source, version) | E | QuantStudio™ Real-Time PCR Software v1.1 (Life technologies), GraphPad Prism V.6 |
| C_q_ or raw data submission using RDML | D | - |

All essential information (E) must be submitted with the manuscript. Desirable information (D) should be submitted if possible.
